# Microvascular dysfunction following cardiopulmonary bypass plays a central role in postoperative organ dysfunction

**DOI:** 10.3389/fmed.2023.1110532

**Published:** 2023-02-14

**Authors:** Shawn Kant, Debolina Banerjee, Sharif A. Sabe, Frank Sellke, Jun Feng

**Affiliations:** Cardiothoracic Surgery Research Laboratory, Department of Cardiothoracic Surgery, Rhode Island Hospital, Lifespan, Providence, RI, United States

**Keywords:** microvasculature, cardiac surgery, vasomotor tone, ischemia-reperfusion, organ damage

## Abstract

Despite significant advances in surgical technique and strategies for tissue/organ protection, cardiac surgery involving cardiopulmonary bypass is a profound stressor on the human body and is associated with numerous intraoperative and postoperative collateral effects across different tissues and organ systems. Of note, cardiopulmonary bypass has been shown to induce significant alterations in microvascular reactivity. This involves altered myogenic tone, altered microvascular responsiveness to many endogenous vasoactive agonists, and generalized endothelial dysfunction across multiple vascular beds. This review begins with a survey of *in vitro* studies that examine the cellular mechanisms of microvascular dysfunction following cardiac surgery involving cardiopulmonary bypass, with a focus on endothelial activation, weakened barrier integrity, altered cell surface receptor expression, and changes in the balance between vasoconstrictive and vasodilatory mediators. Microvascular dysfunction in turn influences postoperative organ dysfunction in complex, poorly understood ways. Hence the second part of this review will highlight *in vivo* studies examining the effects of cardiac surgery on critical organ systems, notably the heart, brain, renal system, and skin/peripheral tissue vasculature. Clinical implications and possible areas for intervention will be discussed throughout the review.

## 1. Introduction

Open-heart operations are among the most frequently performed operations in the United States. Indeed, prior to the COVID-19 pandemic, over 350,000 coronary artery bypass grafting (CABG) operations were performed annually in the US (1). For the past several decades, most open-heart operations, including CABGs, have been performed using cardioplegia and cardiopulmonary bypass (CPB), which arrests cardiac contractility to facilitate surgery while maintaining systemic organ perfusion. Although techniques for cardiac surgery, cardioplegia, and cardiopulmonary bypass have evolved significantly over the years, the associated stress response and inflammation remain major drawbacks.

Many studies in animal models and patients have shown that cardiac operations, especially when involving cardioplegia and cardiopulmonary bypass, induce extensive vascular dysfunction (2). This dysfunction affects large- to medium-sized vessels and the microcirculation, the terminal vascular network of the systemic circulation consisting of a vast array of microvessels < 200 um in diameter. Microvessels can be further subdivided into arterioles, capillaries, and venules, all of which have important roles in maintaining organ function. Arterioles are the primary site of resistance within the vascular network. Thin-walled capillaries are the primary sites of nutrient and gas exchange in the vascular beds that supply every organ system. Finally, venules complete the process of nutrient and gas exchange and begin the process of transporting nutrient-poor, deoxygenated blood from peripheral organ systems back to the heart. Acting in concert, each element of the microcirculation plays a central role in regulating tissue and organ perfusion (3).

Microvascular dysfunction after cardiac surgery is characterized by altered myogenic and vasomotor tone as well as generalized endothelial dysfunction that, in combination, clinically manifest as systemic hypotension and organ damage (2, 4). For example, intraoperative and postoperative inflammation in the microcirculation triggers leukocyte activation, initiating the coagulation cascade in venules. Alternatively, activation of the coagulation cascade in capillaries restricts available surface area for diffusion, resulting in impaired nutrient and gas exchange. Patients may require vasopressors, aggressive intravenous electrolyte repletion, or further escalation in care postoperatively to overcome these clinical consequences.

Understanding the pathogenesis and clinical impact of micro- and macrovascular dysfunction following cardiac surgery is imperative for optimizing surgical technique and postoperative management to reduce morbidity and mortality. This review will begin with a survey of *in vitro* studies that examine the cellular mechanisms of microvascular dysfunction during and after cardiac surgery, paying particular attention to alterations in vascular responsiveness to vasoactive mediators, endothelial activation, and the consequences of inflammation with respect to endothelial injury and barrier integrity. The next section will discuss *in vivo* studies that examine the effects of ensuing microvascular dysfunction on critical organ systems, chiefly the kidneys, brain, lungs, and skin/peripheral tissue vasculature.

## 2. Mechanisms of microvascular dysfunction following cardiac surgery: *In vitro* studies

### 2.1. Dysregulated responses to vasoactive agonists

**FIGURE 1:**
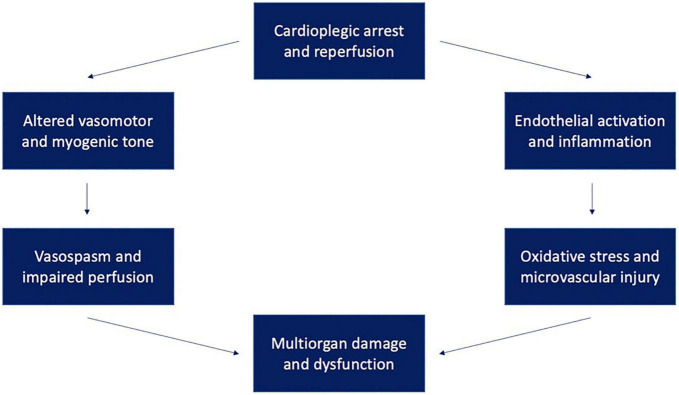
Ischemia-reperfusion injury following cardioplegic arrest may lead to multisystem dysfunction through multiple mechanisms. These mechanisms include endothelial activation and inflammation leading to oxidative stress and altered vasomotor and myogenic tone leading to vasospasm and organ malperfusion.

Figure 1 provides a framework for conceptualizing microvascular dysfunction following cardiac surgery. Extensive research has been done to characterize changes in microvascular responsiveness to vasoactive agonists following cardiac surgery. These agents include neuromodulators, hormones, and endothelium-derived factors that regulate vasomotor tone. Dysfunctional vasomotor tone manifests differently in different vascular beds, triggering reduced vascular resistance in some (e.g., skeletal muscle and peripheral circulation) and increased propensity for vasospasm in others (cardiac, pulmonary, mesenteric, and cerebral vascular circulation) (3). Aberrant vasomotor control in vessel beds supplying major organs contributes to impaired organ perfusion after cardiac surgery, which in turn promotes organ damage.

#### 2.1.1. Neuromodulator systems

Serotonin is a critical mediator of vasomotor tone, and functions predominantly as a vasoconstrictor through the action of the 5HT2A receptor (5). However, serotonin may also act as a vasodilator in certain vascular beds, such as human skeletal muscle arterioles, through action of 5HT2B and 5HT7 receptors (5). Variable changes in serotonergic activity after CPB in different vascular beds may contribute to differing degrees of postoperative organ injury across different systems. For example, studies of human peripheral microvessels dissected from harvested skeletal muscle tissue before and after cardiac surgery involving CPB showed decreased *in vitro* vasomotor responses to serotonin after CPB, with no changes in receptor expression (6). These findings were replicated in the coronary microcirculation through vessels harvested from atrial tissue of patients undergoing cardiac surgery with CPB, with one key exception: in coronary vessels, authors found increased 5HT1B receptor mRNA and protein expression after surgery (7). Furthermore, atrial coronary arterioles from patients undergoing CABG showed increased serotonin-induced microvascular contraction which correlated with increased cyclooxygenase 2 (COX2) protein expression; COX2 inhibition blocked serotonin-induced human coronary microvascular contraction following acute myocardial ischemia (8, 9). Turning to the pulmonary system, swine pulmonary microvessels showed increased contractile responses to serotonin following CPB (10).

Cardiac surgery affects microvascular responsiveness to norepinephrine. Much like serotonin, norepinephrine may have vasodilatory or vasoconstrictive effects depending on the specific receptor involved. Norepinephrine is an important vasoconstrictor in many vascular beds, primarily through action of the alpha-1 adrenergic receptor (11). Meanwhile, norepinephrine may act as a vasodilator through action of beta-adrenergic receptors, in locations such as the myocardial microvasculature (beta-1 receptors) and skeletal muscle vasculature (beta-2 receptors). Changes in adrenergic activity following CPB have predominantly been studied thus far in the coronary and peripheral microcirculations. In the coronary circulation, *in vitro* studies of human coronary arterioles harvested before and after CPB show diminished contractile responses to the alpha-1 agonist phenylephrine, with no differences in alpha-1A or alpha-1B protein expression in harvested tissue (12). In the peripheral circulation, *in vitro* studies of skeletal muscle microvessels from patients before and after CPB showed diminished responsiveness to the beta-adrenergic receptor agonist isoproterenol after surgery (13). No significant changes in total protein expression of any subset of beta-adrenergic receptors were detected (13).

Cardiac surgery may affect activity of neuropeptide Y, another vasoactive neuromodulator. Neuropeptide Y receptor is expressed in human myocardial tissue, primarily in the right atrium (14, 15). Neuropeptide Y signaling has largely been studied in the coronary microcirculation, while further research will be required to elucidate any CPB-driven changes in other vascular beds. In coronary and atrial circulations, neuropeptide Y promotes coronary arteriolar vasoconstriction through action of Y1, Y2, and Y5 receptors (16). Y1 receptors may also mediate action of neuropeptide Y in the peripheral vasculature (17). Results from several studies suggest gender variations in distribution of atrial neuropeptide Y receptor following CPB. One study found that mRNA levels of neuropeptide Y1 and Y5 receptors were significantly lower after cardiac surgery in males, while neuropeptide Y, Y1 receptor, and Y5 receptor levels were significantly higher after cardiac surgery in females (18). These differences were not reflected in intercostal muscle arterioles of patients undergoing CPB, as no significant differences were found in arterial microvascular responsiveness to neuropeptide Y agonists before and after surgery (19).

Finally, cardiac surgery has been shown to influence microvascular responses to the neuromodulator vasopressin. Vasopressin stimulates vascular smooth muscle constriction through V1 receptors and is often used in clinical practice to treat postoperative hypotension and vasoplegia (20, 21). As was the case with norepinephrine, cell-and molecular level analyses of CPB-driven changes in vasopressin activity and responsiveness have largely been studied in the coronary and peripheral microcirculations. Coronary arterioles harvested from right atrial tissue of patients undergoing CPB exhibit significantly increased contractility postoperatively (12). Furthermore, postoperative V1A receptor protein levels were significantly increased compared with preoperative values (12). In contrast, human skeletal muscle arterioles showed decreased vasopressin responsiveness after CPB, suggesting that activation of particular biochemical pathways may be organ-specific (12). Additional work is required to investigate whether these changes are also present in other microcirculations perfusing other critical organs, such as the renal or pulmonary microcirculations.

Altogether, cardiac surgery significantly alters microvascular responses to key neuromodulators, including serotonin, norepinephrine, neuropeptide Y, and vasopressin.

#### 2.1.2. Endothelium-derived agonists

Akin to neuromodulators, a variety of endothelium-derived vasomodulators exhibit altered activity and/or microvascular responsiveness after cardiac surgery, which may also contribute to impaired organ perfusion and organ damage across various key systems. One major endothelial agent is thromboxane A2, a product of endothelial cell membrane arachidonic acid metabolism (22). Thromboxane A2 causes vasoconstriction through its action on vascular smooth muscle thromboxane A2 receptors (22). Thromboxane A2 has been extensively studied in the human coronary microcirculation, where coronary arterioles from patients undergoing cardiac surgery harvested before and after cardioplegic ischemia-reperfusion exhibited attenuated contractile responses to thromboxane A2 analog U-46619 (23). No significant differences were found in the expression of thromboxane A2 receptors before and after cardioplegic ischemia-reperfusion (23). It remains an open question to what extent similar changes may occur in other organ beds.

Endothelin-1 is a potent vasoconstrictor, exerting its action through ETA receptors on vascular smooth muscle cells, and has been studied in coronary and peripheral microcirculations (24). Studies of human skeletal muscle arterioles dissected from tissue harvested from patients before and after cardiac surgery involving cardiopulmonary bypass show that *in vitro* contractile responses to endothelin-1 are significantly decreased after bypass, while total protein levels of ETA and ETB do not change (25). Similar results were found in human coronary arterioles harvested from patients before and after cardiac surgery involving CPB (26). Curiously, some studies have shown that plasma endothelin-1 content increases up to 85% postoperatively, suggesting that decreased microvascular responsiveness to endothelin-1 may be a function of downstream effectors. Finally, given the known role of endothelin-1 in pulmonary microvascular remodeling in pulmonary vascular disease, investigating pulmonary microvascular endothelin-1 responsiveness following CPB may provide very interesting results.

The vascular endothelium also produces many vasodilatory substances, such as nitric oxide, prostacyclin, and endothelium-derived hyperpolarizing factor (EDHF), all of which may be affected by cardiac surgery. Nitric oxide is also known as endothelial-derived relaxing factor (EDRF) and is a byproduct of arginine metabolism by nitric oxide synthase, an enzyme constitutively expressed in endothelial cells. Nitric oxide, in turn, triggers vasodilation through a guanylyl cyclase-protein kinase G signal transduction pathway (27). Three nitric oxide synthase isoforms have been identified: endothelial nitric oxide synthase (eNOS), inducible nitric oxide synthase (iNOS), and neuronal nitric oxide synthase (nNOS). Of these, eNOS and iNOS are of particular interest in microvascular dysfunction following CPB, although the specific effects of CPB on each remain a matter of debate. One *in vitro* study by Toprak et al. of internal mammary arteries taken from patients undergoing CABG with CPB found significantly increased eNOS immunoreactivity postoperatively, with no significant changes in iNOS (28). Meanwhile, a rat model of CPB studied by Hayashi et al. observed increased iNOS activity following CPB (29). Other *in vitro* studies in a variety of animal models, including dogs, pigs, and mice, have shown that eNOS is decreased in coronary and mesenteric microcirculations following cardiac surgery involving CPB (30, 31). Mayers et al. found increased plasma nitric oxide levels in a dog model of cardiopulmonary bypass, along with increased calcium-independent nitric oxide synthase activity (32).

In addition, increased free radical generation due to the systemic pro-inflammatory state induced by CPB may also affect nitric oxide levels (33). Nitric oxide can react with free radicals in several different ways. One way that nitric oxide can react with free radicals is through a process called “scavenging.” In this process, the nitric oxide molecule donates an electron to the free radical, effectively neutralizing it and preventing it from causing damage to cells or tissues. Nitric oxide can also react with certain types of free radicals to form other reactive nitrogen species, such as peroxynitrite or dinitrogen trioxide, which can have a variety of effects on cells and tissues depending on the specific circumstances.

Endothelium-derived hyperpolarizing factor (EDHF), another important vasodilator, plays a central role in an endothelium-dependent, calcium-sensitive signaling pathway that ultimately results in vascular smooth muscle hyperpolarization and vasodilation (34, 35). The ion channels responsible for driving vasodilation in the EDHF pathway are small conductance calcium-activated potassium channels, also known as SK channels (34–37). Extensive research has shown that cardiac surgery induces changes in EDHF pathways across the microvasculature. For example, human coronary microvessels harvested following CPB showed decreased vascular smooth muscle relaxation in response to SK channel activation when compared to vessels harvested before surgery, despite no changes in SK channel gene/protein expression (38, 39). *In vitro* studies of human skeletal muscle arterioles mirrored these results: SK channel activity is reduced following CPB independent of alterations in SK channel polypeptide levels (37).

Hence cardiac surgery has significant effects on microvascular responses to endothelium derived agonists, including vasoconstrictors such as endothelin-1 and thromboxane A2, and vasodilators such as nitric oxide, prostacyclin, and EDHF.

### 2.2. Inflammation and endothelial activation

#### 2.2.1. Overview

Cardiac surgery provokes a strong systemic inflammatory response that has numerous consequences for the microvasculature. Initiating factors for inflammation include exposure of blood to the foreign material of the CPB circuit and ischemia-reperfusion injury, both of which result in increased expression of inflammatory cytokines and generation of reactive oxygen species (40). Studies have demonstrated activation of the complement system and coagulation cascades as blood passes through the CPB circuit. For example, plasma C3a levels were more than five times higher at the end of CPB than the start; this same study also found significant neutrophilia following bypass (41). In addition, plasma thrombin levels have been shown to be significantly increased following CPB (42). Likewise, a meta-analysis of coagulation parameters in patients following cardiac surgery showed that prothrombin time and activated partial thromboplastin time both increased during CPB by up to 33.3 and 17.9%. However, considerable variability was present between different studies (43).

#### 2.2.2. Complement

Complement factors induce synthesis of pro-inflammatory cytokines that promote endothelial activation and chemokine secretion (44). Rossaint et al. investigated the effects of CPB on the leukocyte recruitment cascade, using blood samples from patients undergoing cardiac surgery (45). They found that CPB surprisingly interfered with leukocyte E-selectin-/P-selectin-endothelial ICAM1 interactions and slow rolling (45). However, CPB enhanced neutrophil Mac-1-dependent transmigration across the endothelium into tissue, the final stage of the leukocyte recruitment cascade (45). Chemokine-induced leukocyte migration was significantly enhanced as well, which explains the overall increased leukocyte recruitment during and after CPB (45). Use of leukocyte-reduced blood during cardioplegic ischemia-reperfusion in porcine models appears to promote improved postoperative myocardial perfusion and coronary and cerebral vascular function, suggesting a role for leukocyte-mediated microvascular injury following cardiac surgery (46).

The terminal complement components C5b-9, which form the membrane attack complex, can cause direct damage to the vascular endothelium, as demonstrated in a porcine model of CPB (47). The CPB pump generates shear forces that cause mechanical damage to erythrocyte cell membranes (48), thereby increasing vulnerability to the membrane attack complex. Tofukuji et al. also showed that treatment with a C5a monoclonal antibody reduced endothelial dysfunction as evidenced by endothelium-dependent relaxation responses to ADP and substance P in mesenteric arterioles (47). Other studies have found similar results in other vascular beds, such as swine pulmonary arteries (49). Hence complement activation and membrane attack complex formation are important mediators of inflammatory microvascular damage.

#### 2.2.3. Arachidonic acid metabolism

Arachidonic acid metabolism, another significant inflammatory pathway, is also affected by cardiac surgery. Two critical enzymes that mediate key steps of arachidonic acid metabolism are cyclooxygenase (COX) 1 and 2. COX1 is a housekeeping enzyme, constitutively expressed in most cells. In contrast, COX2 is inducible, with expression increasing in response to endothelial activation and exposure to inflammatory cytokines (50). COX activity also provides a link between inflammation and modulation of vasomotor tone as two key vasoactive mediators, thromboxane A2 and prostacyclin, are both byproducts of arachidonic acid metabolism (thromboxane A2 was discussed extensively earlier).

*In vitro* studies show that inflammation and endothelial stress upregulate COX2 expression in human vascular endothelial cells. This correlates with increased PGI2 and decreased thromboxane A2 production, altering the balance of vasodilation (mediated by PGI2) versus vasoconstriction (mediated by thromboxane A2) in the microvasculature and increasing proclivity for vasospasm (51–53). Looking specifically at cardiac surgery, CPB increases COX2 expression throughout the microvasculature in a manner consistent with inflammation-induced endothelial injury and activation during ischemia-reperfusion (54). Furthermore, experiments in coronary microvessels harvested from patients before and after CPB showed enhanced post-bypass bradykinin-induced relaxation responses, along with increased COX2 protein expression (55). Other studies have shown that inhibiting COX2 following CPB reduces vasoconstriction, not vasodilation, suggesting that the net effect of increased COX2 activity may be increased vasoconstriction (56). Ultimately, what is clear is that arachidonic acid metabolism, and COX enzyme action in particular, play an important role in inflammatory endothelial injury in the microvasculature during cardiac surgery.

#### 2.2.4. Ischemia-reperfusion and free radicals

Both inflammation and ischemia-reperfusion during cardiac surgery in combination lead to generation of free radicals, which when derived from oxygen, are called reactive oxygen species (ROS) (57). First, increased neutrophil activation as blood passes through the extracorporeal CPB circuit enhances NADPH phagosome oxidase activity, leading to increased production of superoxide (58, 59). CPB also increases neutrophil elastase production (60). Next, cardioplegic ischemia-reperfusion disrupts normal tissue blood flow and oxygenation, temporarily interrupting aerobic metabolism in microvascular endothelial cells and perfused tissue. Certain organs, like peripheral vasculature and their end-organs are more resistant to temporary changes in tissue oxygenation than others—including vasculature supplying cardiac or brain tissue, which exhibit functional impairment within minutes of ischemia. Even though cardioprotective refinements over the past several decades have improved outcomes significantly, ischemia-reperfusion injury remains a persistent adverse effect of CPB (61).

**FIGURE 2:**
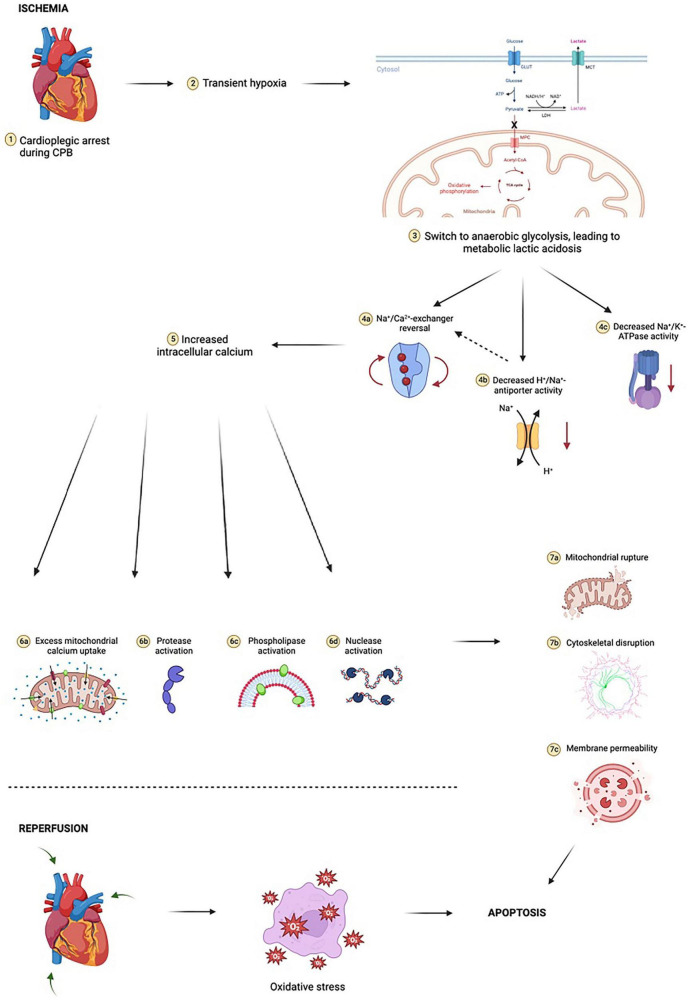
Ischemia-reperfusion injury is complex and multifactorial. At the cellular level, initial transient hypoxia due to cardiopulmonary bypass (CPB) leads to a switch from mitochondrial oxidative phosphorylation to anaerobic glycolysis, with a marked reduction in ATP yield. In poorly oxygenated tissues, pyruvate (the end-product of glycolysis) is shunted away from mitochondria and instead undergoes conversion to lactate by cytosolic lactate dehydrogenase (LDH). The resulting lactic acidosis in combination with high extracellular potassium and hypothermia due to cold cardioplegia inhibits the activity of Na^+^/K^+^-ATPase, an enzyme critical for maintaining physiologic resting membrane potential of cardiomyocytes close to the Nerst equilibrium potential of potassium (approximately –85 to –90 millivolts). Metabolic acidosis dually increases H^+^/Na^+^-antiporter activity, resulting in a small but notable increase in intracellular sodium, further exacerbating the sodium “window current” phenomenon during hyperkalemic cardioplegia. These electrochemical events drive the new membrane potential closer to –50 millivolts. The Na^+^/Ca^2+^-exchanger begins to operate in reverse, moving three Na^+^ ions out of the cardiomyocyte for every Ca^2+^ ion in. Voltage-gated slow calcium channels also begin to activate, leading to further calcium influx. This myocardial calcium loading in turn activates proteases, nucleases, and phospholipases, resulting in phospholipid membrane degradation, organelle destruction, accumulation of catabolic byproducts, compromised ultrastructural integrity of sarcolemma membrane integral to calcium homeostasis, and accelerated depletion of intracellular ATP stores. Prolonged exposure to ischemia increases mitochondrial calcium uptake through reversal of mitochondrial Na^+^/Ca^2+^-exchanger in a manner akin to reversal of cardiomyocyte Na^+^/Ca^2+^-exchanger. This leads to critical and irreversible damage to mitochondria. Following reperfusion, mitochondria have reduced capacity for neutralizing reactive oxygen species (ROS) and contribute to sustained oxidative stress by way of ROS production. This mitochondrial oxidative stress occurs in both cardiomyocytes as well as in the microvasculature, compounding the effects. Cytosolic cytochrome c is implicated in activating caspases involved in intrinsic apoptotic pathways. Reperfusion results in additional cardiac injury independent of ischemic insult which manifests as dysrhythmia, myocardial stunning, and myocardial necrosis. Notably, concurrent oxidative stress within the microvasculature results in microvascular dysfunction, further compounding cardiac injury. Figure created using BioRender.com.

Ischemia during cardiac surgery triggers a cascade of events that result in increased oxidative stress (62; Figure 2). First, oxygen depletion disrupts oxidative phosphorylation, the cornerstone of aerobic metabolism, and forces cells to rely exclusively on anaerobic glycolysis. Anaerobic metabolism is considerably less efficient at generating ATP than aerobic metabolism, and results in increased lactic acid generation, thus lowering intracellular pH and creating a suboptimal environment for the function of many intracellular enzymes. Low intracellular ATP interferes with the ability of the Na^+^-K^+^ ATPase to transport Na^+^ and K^+^ across the cell membrane, ultimately disrupting resting cell membrane potential. With prolonged exposure to ischemia, intracellular calcium levels rise in ischemic cells, leading to activation of phospholipases, nucleases, and proteases that begin degrading organelles, cytoskeletal protein, and cellular DNA. One of the organelles particularly sensitive to calcium is the mitochondrion, as calcium promotes opening of mitochondrial permeability transition pores (MPTPs) that allow pro-apoptotic oxidative mitochondrial enzymes, such as cytochrome c, to leak into the cytosol and increase oxidative stress.

Reperfusion after ischemia initiates yet another biochemical cascade that results in ROS generation (Figure 2). Abrupt restoration of blood flow to hypoxic tissue and reentry of oxygen into vascular endothelial cells stimulate pathways involving enzymes such as NADPH oxidase, xanthine oxidase, and nitric oxide synthase. These pathways generate primary ROS (e.g., superoxide anions) and secondary ROS (such as hydroxyl radicals, peroxynitrite, and hypochlorite) (63). Notably, red blood cell destruction due to membrane attack complex formation also results in leakage of free hemoglobin into the blood, which in the presence of hydrogen peroxide, may act as a peroxidase.

Peroxynitrite disrupts nucleic acids, proteins, and lipids. Cell and mitochondrial membrane damage due to ROS promotes apoptosis, which in the microvasculature results in extensive endothelial dysfunction and cell death (64). ROS may also further activate proapoptotic and proinflammatory pathways, such as MAPK and NFkB signaling, through nitrosylation, carbonylation, disulfide bond formation, and glutathionylation (63). An *in vitro* model of cardioplegic hypoxia-reoxygenation using mouse small coronary arteries and endothelial cells showed that application of the mitochondria-targeted antioxidant, Mito-Tempo, protected vascular relaxation responses after cardioplegic hypoxia-reoxygenation (65). Overall, Mito-Tempo reduced mitochondrial ROS overload, and also protected endothelial SK channel currents, suggesting a link between ROS and SK channel dysfunction after cardiac surgery. Further research in humans will be needed to verify the translational potential of mitochondrial ROS inhibition as a tool for mitigating coronary microvascular dysfunction after CPB. Altogether, ischemia-reperfusion injury, and free radicals/ROS generated in the process, serves as a critical mechanism of microvascular damage following cardiac surgery.

## 3. Postoperative vascular dysfunction and organ damage: *In vivo* studies

### 3.1. Vascular dysfunction in renal injury

#### 3.1.1. Overview

Acute kidney injury (AKI) remains a common and well-established postoperative complication of cardiac surgery, in particular cardiac surgery involving CPB. AKI is defined as an abrupt decline in kidney function due to renal impairment (66). There are many causes of AKI, which may fall under one of three broad categories: pre-renal, intrinsic renal, and acute post-renal disease. Two of these categories, pre- and post-renal, reflect the consequences of extrarenal processes, while intrinsic renal disease represents pathology associated with glomeruli, interstitial vessels, or renal tubules. Pre-renal causes of AKI are linked to decreased renal perfusion, which may arise from a variety of factors including intravascular volume depletion, medication effect, sepsis, heart failure, or other causes of shock (67). Intrinsic renal causes of AKI include acute tubular necrosis due to infection, drugs, or toxins; acute interstitial nephritis, and glomerulonephritis. Postrenal causes of AKI center on urinary tract obstruction. AKI may result from a combination of causes in these three categories.

The diagnosis of AKI largely involves interpretation of serum creatinine, often in the context of a decline in urine output (68, 69). According to the Kidney Disease Improving Global Outcomes classification (KDIGO), AKI can be categorized from stage I (less severe) up to stage III (most severe) depending on serum creatinine, glomerular filtration rate (GFR), and/or need for renal replacement therapy (69).

The renal system is exquisitely vulnerable to microvascular dysfunction and subsequent hypoxemia, which drives AKI and subsequent macrohemodynamic pathology. First, the kidneys consume nearly 25% of total cardiac output, an amount necessary for sustaining glomerular filtration (70). Renal oxygen consumption per gram of tissue is also extraordinarily high despite relatively poor renal oxygen extraction; only the heart itself consumes more (2.7 mmol/kg/min for the kidneys vs. 4.3 mmol/kg/min for heart) (70). The most significant contributor to high renal oxygen demand is tubular transport (e.g., oxygen costs of ion reabsorption along the tubular network).

Renal microvascular oxygen supply and consumption are regulated by a variety of factors, chief among which are nitric oxide, angiotensin II, and HIF-1alpha (71–73). Nitric oxide-induced vasodilation augments renal blood flow and oxygen delivery, and decreased nitric oxide levels may contribute to high renal oxygen demand in the setting of chronic kidney disease (71). Angiotensin II induces renal vasoconstriction, reducing renal oxygen delivery and increasing renal oxygen demand; pathologic/hyperactive angiotensin II is also thought ot play a role in ischemic renal disorders such as hypertension and chronic kidney disease (72). HIF-1alpha can activate inducible nitric oxide synthase and VEGF to promote renal oxygen delivery and angiogenesis (73).

**FIGURE 3:**
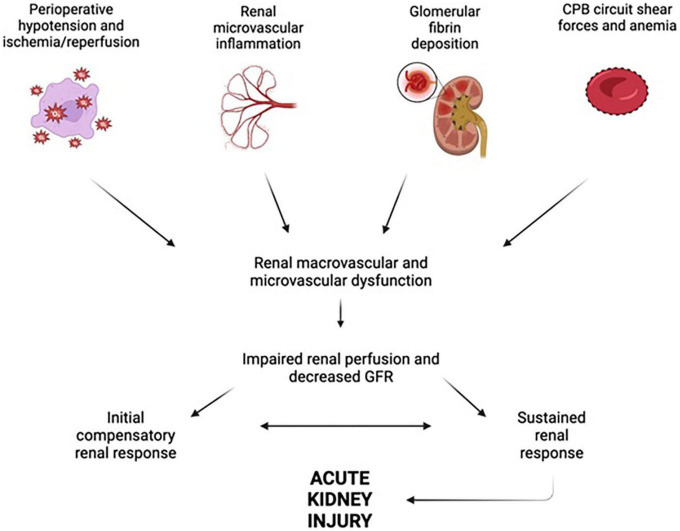
Factors leading to renal dysfunction during and after cardiopulmonary bypass. Perioperative hypotension, renal microvascular inflammation, glomerular fibrin deposition, and cardiopulmonary bypass (CPB) circuit shear forces all act in concert to impair renal perfusion and glomerular filtration rate (GFR), ultimately leading to acute kidney injury. Figure created using BioRender.com.

The incidence of AKI following cardiac surgery has been reported to be as low as 3.4% and as high as 40% (74–76). Indeed, longer duration of CPB is related to increased likelihood of postoperative AKI and acute renal failure requiring renal replacement therapy (77–80). Many factors have been shown to contribute to this, including hypotension and hemodynamic disturbances, inflammation, and nephrotoxins; Figure 3 provides an overview of factors influencing AKI after cardiac surgery.

#### 3.1.2. Perioperative hypotension and AKI after CPB

Of these factors, the most well-studied is hemodynamic instability due to sustained perioperative hypotension (81). In one retrospective study examining AKI after cardiac surgery, cardiogenic hypotension was determined to be a major factor underlying AKI in 46.3% of patients (82). Recovery from anesthesia is one potential cause of postoperative hypotension (83). However, a more significant factor driving sustained hypotension after CPB is likely vasoplegia, with a pathophysiology very similar to that of sepsis-induced vasodilatory shock (84). Indeed, hallmarks of vasoplegia include normal or augmented cardiac output in the setting of low systemic vascular resistance, leading to organ hypoperfusion (84, 85).

Potential causes of vasoplegia include altered microvascular responsiveness to vasomodulators such as serotonin, endothelin-1, vasopressin, EDHF, and thromboxane A-2, all of which were discussed extensively earlier. Inflammation and pathologic endothelial activation as blood is exposed to the CPB circuit may also contribute to abnormal postoperative microvascular myogenic tone and vasoplegia. Duration of postoperative vasoplegia and extent of microvascular recovery can be widely variable between patients; however, a large portion of patients require at least temporary period of vasopressor support to maintain blood pressure in the immediate postoperative period (83, 85).

Another retrospective study determined that duration of intraoperative hypotension was significantly associated with development of stroke, AKI, and postoperative mortality (*p* = 0.032) (86). There is no universally accepted definition of intraoperative hypotension. Most blood pressure value thresholds have traditionally been chosen based on what is required to maintain adequate cerebral perfusion (86). However, the lower limits of cerebral autoregulation may vary wildly, anywhere between 40 to 160 mmHg; this implies a wide range of flexibility in determining MAP during cardiac surgery involving CPB (86). Causes of intraoperative hypotension during cardiac surgery include decreased venous return from cardiac manipulation, arrhythmias, and decreased systemic vascular resistance (incipient vasoplegia, which is magnified in the postoperative period (87). However, the effect of intraoperative hypotension and reduced renal perfusion pressure during cardiac surgery on AKI may not be as clear-cut as initially thought. For example, a recent randomized controlled trial compared incidence of postoperative AKI in 90 patients who underwent cardiac surgery involving CPB, with the patients divided into a high arterial pressure group (defined in this study as MAP during CPB greater than 60 mmHg) and a control group. Total postoperative incidence of AKI was 41%, and the investigators found no significant differences between the control and high MAP groups (88). Similar findings were reported in a multicenter retrospective cohort study conducted by Kotani et al., who examined the impact of mean perfusion pressure deficit, defined as difference between MAP and central venous pressure (CVP), on AKI in over 700 adult patients admitted to 14 ICUs after elective cardiac surgery (89). Overall, no clear association between relative hypotension and AKI progression post-cardiac surgery was appreciated (89).

#### 3.1.3. Microvascular inflammation and AKI after CPB

Another potential contributor to acute kidney injury after cardiac surgery is the systemic pro-inflammatory state induced by CPB during on-pump surgery. Zhang et al. examined associations between two key inflammatory cytokines, IL-6 and IL-10, and AKI in a six-center study of adult cardiac surgery patients (90). IL-6 and IL-10 levels increased after cardiac surgery, and higher postoperative levels of IL-6 and IL-10 were significantly associated with higher risk of AKI in a dose-dependent manner (90). A separate multicenter study of adult cardiac surgery patients conducted by Moledina et al. found that cardiac surgery led to significant increases in Th1- and Th2-dependent cytokines, most notably interferon-gamma, IL-4, and IL-13 levels (91). Patients with higher levels of IL-4, IL-13, and interferon-gamma had higher odds of postoperative AKI and mortality (91).

Intraoperative inflammation has been linked to exposure of blood to the CPB extracorporeal circuit, ischemia-reperfusion injury, and oxidative damage (92). Ischemia-reperfusion in particular triggers production of ROS through various mechanisms involving mitochondrial damage, endothelial activation, and plasma membrane breakdown (93, 94). ROS upregulate many pro-inflammatory transcription factors such as NFkB, which (1) promote expression of inflammatory cytokines, chemokines, and inflammasome formation, and (2) regulate activation and differentiation of T cells and innate immune cells (95). Immune cells, activated by cytokines, demarginate out of systemic circulation and extravasate into different organ systems including the renal parenchyma, stimulating parenchymal inflammation, tubulo-interstitial fibrosis, and AKI (96). It is important to note here that a similar cascade of microvascular inflammation is a likely contributor to postoperative organ damage in other organ systems, as will be discussed in the following sections.

#### 3.1.4. CPB shear forces, hemoglobin, and AKI

Cardiopulmonary bypass (CPB) also exposes blood to a variety of shear forces in the extracorporeal circuit (pump, suction catheters, filters) that lyse erythrocytes and result in free hemoglobin spilling into the systemic circulation (97, 98). Circulating haptoglobin binds a portion of free hemoglobin, but haptoglobin stores may become quickly depleted in the setting of large amounts of free hemoglobin. Remaining free hemoglobin is ultimately filtered by the kidneys.

There are several ways in which hemoglobin is nephrotoxic. First, the kidneys sequester a large amount of free iron, and hemoglobin promotes intrarenal conversion of ferrous hemoglobin to ferric hemoglobin and accumulation of hemoglobin-cross linked products, which cause precipitation of Tamms Horsfall proteins in the collecting system, leading to intrinsic acute tubular injury (99). In addition, hemoglobin facilitates free radical production in the kidneys, along with enhanced expression of heat shock proteins (100). Free radicals stimulate lipid peroxidation and catalyze formation of deleterious ROS. In a case control study comparing 10 cardiac surgery patients with AKI to 10 risk-matched controls, investigators found that patients who developed AKI after cardiac surgery had twice as much plasma-free hemoglobin than controls (101). Curiously, the iron chelating agent deferoxamine was shown to decrease lipid peroxidation during CPB (102). Although some studies have demonstrated promising initial results for the use of iron chelation as a therapeutic tool to prevent AKI, specific studies examining whether intraoperative iron chelation may be protective against cardiac surgery-induced AKI are required to provide greater clarity (103).

Anemia due to CPB-related hemodilution may itself influence postoperative AKI given the exquisite sensitivity of the kidney to adequate tissue oxygenation (104). Preoperative anemia has been associated with increased incidence of AKI post-CABG with CPB (105). Moreover, a critical perioperative hemoglobin nadir of < 8.05 g/dL has been associated with AKI, acute myocardial infarction, and stroke following CPB (106). Meanwhile, Cao et al. report a critical hemoglobin threshold of > 9 g/dL with respect to incidence of AKI following CPB; although curiously, the impact of intraoperative RBC transfusions on AKI to maintain hemoglobin levels above 9 g/dL was of unclear effectiveness (104).

#### 3.1.5. Pre-existing microvascular dysfunction and kidney injury after CPB

Pre-existing microvascular dysfunction in cardiac surgery patients may influence the likelihood of renal injury due to microvascular dysfunction during and after CPB. For example, anywhere between 30−−40% of patients undergoing cardiac surgery carry a preoperative diagnosis of DM (107). Poorly controlled diabetes/uncontrolled hyperglycemia has numerous deleterious consequences on the microvasculature, including accelerated atherosclerosis, increased capillary basement membrane thickening and extracellular matrix proliferation, and inflammation and oxidative stress resulting in endothelial injury due to advanced glycated end products (108, 109). In the renal system, a combination of diabetic micro and macroangiopathy along with direct hyperglycemic/inflammatory damage to the renal parenchyma produces characteristic histopathological changes including intimal hyalinosis of afferent and efferent arterioles leading to glomerular hyperfiltration, and ultimately diffuse diabetic glomerulosclerosis (110).

Elevated preoperative hemoglobin A1c and fasting blood glucose levels prior to CABG surgery have been linked with increased frequency of postoperative morbidity and mortality, including postoperative wound infection, bleeding, arrhythmias, and atelectasis (111). Studies specifically examining the relationship between preoperative hemoglobin A1c and postoperative renal morbidity reveal a higher incidence of postoperative AKI in CABG patients with hemoglobin A1c greater than 5.9% (112). Likewise, Wang et al. found that independent of baseline renal or cardiac function, patients with DM were more likely to have AKI after CABG (113). Furthermore, diabetic patients on insulin had increased odds ratio for AKI than diabetic patients on oral glycemic agents (113).

Another important pre-existing condition widely prevalent among cardiac surgery patients is hypertension (114), an unsurprising observation given the strong connection between hypertension and cardiovascular disease writ large. Uncontrolled hypertension induces microvascular dysfunction through abnormal vasomotor tone (enhanced vasoconstrictive and impaired vasodilatory responses) and pathological vascular remodeling (115). Increased vascular stiffness results from hypercontractility of vascular smooth muscle cells, intimal hyperplasia, advential fibrosis, and eventually hypertensive arteriolosclerosis (116).

Hypertensive vasculopathy ultimately affects every vascular bed in the body; however, particularly deleterious effects have been observed in the renal and cerebral microvasculature. In the kidneys, hypertensive arteriolosclerosis compromises blood flow to the kidneys, resulting in tubular atrophy, interstitial fibrosis, glomerular ischemia, and ultimately glomerulosclerosis (117). Despite these well-known renovascular consequences, few studies have directly examined links between preoperative hypertension and AKI following cardiac surgery, although plenty of studies have examined postoperative hypertension following cardiopulmonary bypass (118, 119). One review by Weir et al. found that overall, preexisting systolic hypertension appears to correlate well with postoperative AKI (120). However, further work is required to fully characterize the cellular and molecular-level changes underpinning these observations.

#### 3.1.6. Techniques of perfusion and kidney injury after CPB

Finally, the form of perfusion used during CPB may also influence postoperative kidney injury. For example, use of pulsatile flow during CPB has been increasing with advancements in extracorporeal circuit technology (121). In theory, pulsatile flow more closely approximates physiological blood flow of the human heart and delivers increased mechanical energy to the vascular endothelium (122). The resulting endothelial shear stress is thought to augment release of vasodilatory substances, decrease systemic vascular resistance, and improve organ perfusion (122). With respect to the kidneys, renal tissue perfusion was substantially higher during pulsatile perfusion vs. non-pulsatile in a porcine model of CPB (123).

Milano et al. found that patients undergoing pulsatile perfusion CPB during aortic valve replacement had no significant differences in pre vs. postoperative creatine clearance, while a statistically significant difference was observed in patients undergoing normal perfusion (122). Similar results were found by Natarajan et al. (124). Mohammadzadeh et al. found more extensive dysregulation in BUN and creatinine levels in patients undergoing non-pulsatile CPB (125). In addition, a meta-analysis conducted by Sievert and Sistino of 298 articles found that patients undergoing pulsatile perfusion had significantly higher creatinine clearance postoperatively in the ICU, although there were no significant differences between groups in terms of mean postoperative creatinine levels or BUN (126). Nonetheless, in their conclusion, the authors report that the potential benefit of pulsatile perfusion during CPB with respect to renal preservation should strongly be considered (126).

Overall, AKI is a common postoperative insult following cardiac surgery, and is driven by a variety of forces that harm the renal microvasculature including intraoperative hypotension, renal inflammation and glomerular fibrin deposition, and hemoglobin nephrotoxicity due to red blood cell shearing during cardiopulmonary bypass.

### 3.2. Vascular dysfunction and CNS injury

#### 3.2.1. Overview

A variety of neurological deficits may occur following cardiac surgery involving cardioplegia/CPB. Early iterations categorized such deficits into two broad groups: Type 1 and Type 2 deficits. Type 1 deficits refer to major focal complications including stroke, coma, and hypoxic encephalopathy, while type 2 deficits refer to generalized deteriorations in intellectual function, including confusion, agitation, disorientation, and memory deficits (127–129). The most recent update of the American College of Cardiology/American Heart Association guidelines for CABG operations in 2011 present three overarching types of postoperative neurological deficits: stroke, coma, and general neurocognitive deficits (130). Like the old type 2 grouping, general neurocognitive deficits include features such as postoperative deteriorations in memory and intellectual function without definitive evidence of focal neurologic injury. When comparing a patient’s baseline and postoperative neurocognitive status, the presence of the symptoms on a neurocognitive assessment battery may indicate neurocognitive decline.

In a multicenter prospective study, Roach et al. found that adverse cerebral outcomes (including focal injury, stupor, coma, memory deficits, seizures, and general deterioration in intellectual function) occurred in 6.1% of patients (127). Patients with adverse neurologic outcomes exhibited increased mortality, longer hospitalization, and higher rate of discharge to long-term care facilities (127). El Bardissi et al. analyzed data from the Society of Thoracic Surgeons adult cardiac surgery database between 2000 and 2009, finding an average incidence of post-CABG stroke of 1.3% (131). Moreover, in a prospective MRI study of 127 CABG patients, Nah et al. found that 27.6% showed new cerebral infarcts postoperatively, although most were clinically silent. Clinically evident (symptomatic) stroke was seen in 3.1% of patients (132).

Turning to neurocognitive decline, different studies report varying incidence rates following cardiac surgery at different time periods. For example, in one study of over 200 CABG patients, about 53% of patients experienced cognitive decline (defined as a drop of one standard deviation in test scores measuring any one of four cognitive domains postoperatively vs. preoperatively) at discharge, 36% at 6 weeks, 24% at 6 months, and 42% at 5 years (133). This same study also found that cognitive function at discharge was a good predictor of long-term cognitive function (133). Other studies report incidence rates of postoperative neurocognitive decline may vary anywhere from 10 to 40% 1 to 3 months after cardiac surgery (134).

#### 3.2.2. Emboli during CPB and cerebral vascular dysfunction

**FIGURE 4:**
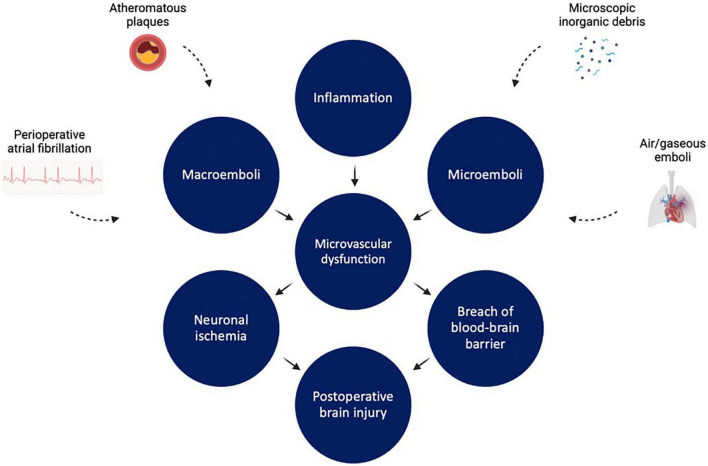
Factors leading to cerebrovascular dysfunction during and after cardiopulmonary bypass. Inflammation, microemboli, and macroemboli all lead to microvascular dysfunction. This in turn leads to neuronal ischemia and disruption of blood brain barrier integrity, resulting in brain injury. Figure created using BioRender.com.

Much like renal injury after cardiac surgery, the pathophysiology of brain injury after cardiac surgery is multifaceted and implicates micro- and macrovascular complications (Figure 4). First, thromboembolic events obstructing the cerebral vasculature play a significant role in post-cardiac surgery stroke and encephalopathy (135). A study of 388 post-CABG stroke patients in New England between 1992 and 2000 showed that embolic strokes accounted for 62.1% of strokes, with the remainder being attributed to other etiologies such as hypoperfusion, hemorrhage, and lacunar infarcts (136). Sources of emboli are many. Regarding macroemoboli, intraoperative ultrasonography of the ascending aorta performed in 500 cardiac surgery patients showed that 68 (13.6%) had significant atheromatous disease of ascending aorta, putting them at high risk for atheroembolism (137). Alternatively, pre or intraoperative atrial fibrillation may also potentiate development of macroemboli due to atrial blood pooling, as suggested by results of another study analyzing data from 3,008 patients who underwent CABG or valve surgery between 2008 and 2012 (138). In this study, the incidence of postoperative stroke in patients with new-onset atrial fibrillation and in patients with preoperative and postoperative atrial fibrillation was significantly higher than in patients with no atrial fibrillation (138).

In contrast to macroemboli, microemboli are generally particulate (e.g., microscopic epicardial fat globules, microscopic inorganic debris) or gaseous (e.g., air emboli). These often enter the CPB circuit during venous cannulation or during perfusionist interventions (e.g., when air is injected into the venous side of the CPB circuit), and can cause significant harm to the vasculature. Gas bubbles in particular can cause significant damage to the vascular beds of all organ systems, inducing inflammation, coagulation, platelet activation, and complement activation (139). In the cerebral vasculature, small gas bubbles less than 15 mm in diameter (a common size for gas microemboli in patients undergoing CPB) may disrupt the blood-brain barrier (140). This exposes the brain to a variety of systemic toxins, alters regulation of cerebral blood flow, and exacerbates a pro-inflammatory microenvironment in the central nervous system (141, 142). Of note, increased duration of CPB has been linked with increased incidence of postoperative cerebral microemboli (143).

Gas microemboli have been implicated in global neurocognitive dysfunction following cardiac surgery (144, 145). Transcranial doppler ultrasonography of 100 patients undergoing cardiopulmonary bypass showed that patients with increased brain microembolic burden had more significant neuropsychological deficits after surgery (146). Borger et al. administered a battery of neuropsychologic tests pre- and postoperatively to 83 patients who underwent elective CABG, and found that patients exposed to more than 10 perfusionist interventions, which correlated with increased burden of gaseous microemboli, exhibited lower mean scores on 9 of 10 neuropsychological tests; Results from Rey Auditory Verbal Learning, Digit Span, and Visual Span achieved statistical significance (144). Finally, an analysis of 102 CABG patients showed a strong inverse correlation between the intensity of intraoperative gaseous microembolic signals (monitored by non-invasive, real-time ultrasound) and performance on postoperative neurocognitive tests (147). Curiously, no definitive link has been found linking duration of CPB and postoperative cognitive impairment (148).

Given these findings, the question arises whether intraoperative interventions to reduce microemboli may mitigate cerebral microvascular damage and improve postoperative neuropsychological function. In a porcine model of CPB, intraoperative hypobaric oxygenation to reduce dissolved gases in the blood and CPB circuit resulted in significant reduction in cerebrovascular gaseous microemboli burden; subsequent histopathologic analysis of brain tissue suggested decreased microvascular injury (149). Gerriets et al. studied whether an intra-aortic filter designed to reduce solid microemboli or a dynamic bubble trap designed to reduce gaseous microemboli affect cognitive functioning 3 months after CABG (150). Transcranial doppler sonography was used to detect signs of microemboli during surgery. Final analysis showed that the dynamic bubble trap group exhibited better executive functioning and short-term memory compared with controls, while there was no difference in cognitive test results between the intra-aortic filter group and controls (150). Total microemboli were lower in the dynamic bubble trap group compared to all other groups (150).

#### 3.2.3. Impact of cerebral hypoperfusion and pre-existing microvascular dysfunction

Beyond macro- and microemboli, cerebral hypoperfusion is another potential mechanism of cerebrovascular injury after cardiac surgery. Autoregulation of vascular tone, influenced by soluble mediators, is critical for maintenance of adequate cerebral blood flow. PaCO_2_ is one such mediator. During cardiac surgery involving cardioplegia and CPB, alpha-stat pH management cools the blood passing through the CPB circuit, resulting in heightened blood CO_2_ solubility and increased pH (151). For this reason, CO_2_ is often added to the CPB circuit in order to normalize PaCO_2_—this in turn facilitates cerebral vasodilation and over time, exhausts autoregulation (135). Cerebral hypoperfusion may have an even greater impact on brain injury during off-pump cardiac surgery, where cerebral venous hypertension may occur secondary to traction on the superior vena cava as the heart is displaced to achieve better access to the coronary arteries (152).

Moreover, in a single photon emission computed tomography (SPECT) imaging study of 82 patients referred for CABG, 75% of scans demonstrated regions of abnormal cerebral perfusion, associated with old age, tobacco use, and diabetes (153). Pre-existing conditions that lead to altered cerebral perfusion are prevalent in patients undergoing cardiac operations, putting these patients at greater risk of cerebrovascular injury in the setting of intraoperative cerebral hypoperfusion during CPB.

For example, as discussed earlier, many patients undergoing cardiac surgery carry a preoperative diagnosis of hypertension, which itself is a well-established cause of extensive micro and macrovascular disease across all vascular beds. Hypertension is associated with pathological cerebrovascular remodeling, resulting in impaired resting cerebral blood flow and higher resting cerebrovascular resistance (154). This also results in impaired cerebral autoregulation, placing the brain at increased vulnerability to ischemia from neurovascular disease. Hypertension may also cause breakdown of the blood-brain barrier through a variety of mechanisms involving inflammation and oxidative stress due to shear forces and endothelial activation (155).

Furthermore, hypertensive small-vessel disease itself is a major cause of deep brain lacunar infarcts, alongside cerebral amyloid angiopathy. Hence unsurprisingly, hypertension is considered critical risk factor for perioperative stroke in patients undergoing cardiac surgery (156). Likewise, hypertensive microvascular disease may be connected to increased likelihood of general neurocognitive impairment after cardiac surgery (134). However, more work will be required to elucidate mechanisms behind any potential connections.

In addition, diabetes has been linked to cerebrovascular disease, largely due to accelerated atherosclerosis, increased edema, and neovascularization in the setting of endothelial damage, oxidative stress, and aberrant inflammatory activation (157) (see previous section for general discussion on diabetic microvascular disease). Similar to hypertension, extensive atherosclerosis in the setting of DM contributes to ischemic cerebrovascular disease and vascular cognitive impairment, increasing risk for cerebral infarction and transient ischemic attacks (157). Indeed, diabetic patients are 2–6 times more susceptible than non-diabetic patients to stroke events (158). Multiple studies have demonstrated that both type 1 and type 2 DM are associated with increased long term risk of stroke following CABG, although much like hypertension, the specific mechanisms underpinning this observation in the setting of cardiac surgery require elucidation (159, 160).

A study of 15 patients at high risk for postoperative stroke who underwent CABG and found that an intraoperative drop in MAP compared to baseline MAP portended a decrease in Mini-Mental Status Examination score postoperatively (161). A separate study examining 98 patients with clinical stroke after cardiac surgery who underwent diffusion-weighted MRI showed that bilateral watershed infarcts were present in 48% of MRIs (162). Further analysis showed that patients with decrease in MAP of at least 10 mmHg intraoperatively were 4.1 times more likely to have experienced bilateral watershed infarcts, as watershed regions in the brain are particularly vulnerable to ischemic stroke in the setting of cerebral hypoperfusion and microvascular dysfunction (162).

#### 3.2.4. Techniques of perfusion and brain injury after CPB

Much like with the renal system, pulsatile perfusion may have a benefit in terms of reduced postoperative brain injury. A systematic review conducted by Ji and Undar found that pulsatile flow significantly improved intraoperative and postoperative blood flow to brain, heart, liver, and pancreas in pediatric and adult patients undergoing cardiac surgery (163). Furthermore, in a pediatric population of patients undergoing CPB to repair congenital heart defects, patients undergoing pulsatile perfusion had lower decreases in regional cerebral oxygen saturation from baseline compared to patients undergoing non-pulsatile perfusion (164). In contrast, non-pulsatile perfusion during CPB has been associated with increased cerebrovascular carbon dioxide reactivity and more profound decreases in cerebral blood flow during periods of hypocapnia (165).

With respect to specific neurocognitive outcomes, one prospective study showed a significantly reduced rate of mild cognitive impairment in patients undergoing pulsatile perfusion during CABG (166). However, a different prospective study of CABG patients by Ozturk et al. found no significant difference in postoperative MMSE scores between pulsatile vs. non-pulsatile perfusion groups (167). Overall, there is no clear consensus regarding whether pulsatile perfusion truly impacts postoperative cognitive decline following CPB, and the matter remains one of extensive debate (168, 169). In addition, there is a surprising lack of research examining whether pulsatile perfusion affects incidence of postoperative ischemic stroke, which is another vital area of investigation.

#### 3.2.5. Role of temperature and brain injury after CPB

Temperature of CPB, chiefly degree of hypothermia, may also have an impact on postoperative CNS injury. A variety of studies have examined the impact of CPB temperature on cerebral oxygenation. One single center trial by Lenkin et al. showed that cerebral tissue oxygen delivery and consumption were significantly higher with normothermic (36.6 degrees Celsius) vs. hypothermic (32 degrees Celsius) CPB (170). However, Kadoi et al. found that regional cerebral oxygenation state decreased with normothermic (> 35 degrees Celsius) vs. hypothermic (30 degrees Celsius) CPB (171). In addition, both normothermic (37 degrees Celsius) and hypothermic (37 degrees Celsius) CPB have been associated with similar degrees of postoperative cerebral edema based on T1-weighted and FLAIR MRI imaging (172).

Unfortunately, the extent to which normothermic vs. hypothermic CPB protects or promotes postoperative brain injury is unclear. A porcine model of deep hypothermic CPB (cooling to 18 degrees Celsius) demonstrated significantly increased cerebral mitochondrial dysfunction compared to pigs undergoing normothermic CPB (173). Likewise, hypothermic circulatory arrest was associated with significantly increased blood-brain-barrier dysfunction compared to normothermic CPB (174). Along this line, some studies, such as that by Grimm et al. found that patients undergoing mild hypothermic (32 degrees Celsius) CPB had significantly worse neurocognitive functioning 1 week after surgery and 4 months after surgery compared to patients undergoing normothermic (37 degrees Celsius) CPB (175). However, by 4 months after surgery, no significant difference could be found (175). Meanwhile, other studies and systematic reviews have reported insufficient evidence to declare any significant differences (176, 177).

#### 3.2.6. Intraoperative NIRS and cerebral injury following CPB

Near-infrared spectroscopy (NIRS) has been investigated as a non-invasive real time tool to monitor oxygenation at the level of the microcirculation in the CNS (178, 179). One single-center prospective study of patients undergoing CPB found that NIRS-directed intraoperative interventions (e.g., increasing MAP, increasing inspired oxygen, correcting bypass cannula) aimed at maintain cerebral saturations at or above baseline resulted in significant improvement in 6 months general neurocognitive function postoperatively, although only length of ICU stay was statistically significant (shorter) in the NIRS group (180). Another single-center prospective trial examining NIRS-directed intraoperative interventions (including fluid challenge, deepening anesthesia, and administration of vasoactive medications) during episodes of cerebral oxygen desaturation (< 60% for > 60 consecutive seconds) during CPB found that 6 months postoperative group mean memory change scores were significantly improved in the intervention group, although overall perioperative outcomes did not differ between intervention and control groups (181). Likewise, a retrospective study evaluating association of intraoperative cerebral oxygen monitoring during cardiopulmonary bypass on postoperative complications found that cerebral oximetry monitoring during cardiothoracic surgery was associated with lower incidence of stroke and overall mortality rate (182).

Curiously, results from systematic reviews examining impact of NIRS-directed intraoperative interventions on neurologic outcomes following cardiopulmonary bypass have been mixed. One review by Serraino and Murphy of 10 randomized trials found no clear evidence to support a significant clinical benefit for cerebral NIRS-based algorithms in cardiac surgery (183). Similarly, a systematic review by Zheng et al. found that while intraoperative reductions in regional cerebral oxygen saturation (rScO2) may be able to identify CPB cannula malposition, only low-level evidence linked low rScO2 with postoperative neurological complications such as stroke or postoperative cognitive decline (184).

Finally, as with the renal system, hemodilution and subsequent anemia during CPB may influence degree of impaired cerebral oxygenation and brain injury. Hemodilution during CPB worsened cerebral infarct size after middle cerebral artery occlusion in rats randomized to CPB with extensive hemodilution (hemoglobin 6 g/dL) vs. controls (hemoglobin 11 g/dL) (185). Moreover, a pig model of CPB investigating the impact of total blood prime resulting in high hematocrit (33%) vs. crystalloid prime resulting in low hematocrit (14%) revealed significantly decreased cerebral oxyhemoglobin and total hemoglobin with low hematocrit by NIRS assessment (186). Addition of hemoglobin-based oxygen carrier attenuated CPB = induced cerebral damage, measured through biomarkers such as neuron-specific enolase, in cerebrospinal fluid and serum of a dog model of CPB (187). Finally, a study evaluating patients undergoing mitral valve replacement under CPB determined a hemoglobin threshold of 9.2–9.4 g/dL for avoiding significant EEG band shifts during CPB (188). Such studies may help influence development of guidelines for maintaining intraoperative hematocrit/hemoglobin levels, for which there are currently no universally accepted standards.

In summary, brain injury following cardiac surgery can manifest in a variety of ways, some hyperacute, such as ischemic/thromboembolic stroke, and others more subtle, such as postoperative neurocognitive decline. Vascular injury due to cerebral hypoperfusion, inflammation, microemboli and macroemboli are all important contributors to postoperative brain injury.

### 3.3. Vascular dysfunction and the pulmonary system

#### 3.3.1. Overview

**FIGURE 5:**
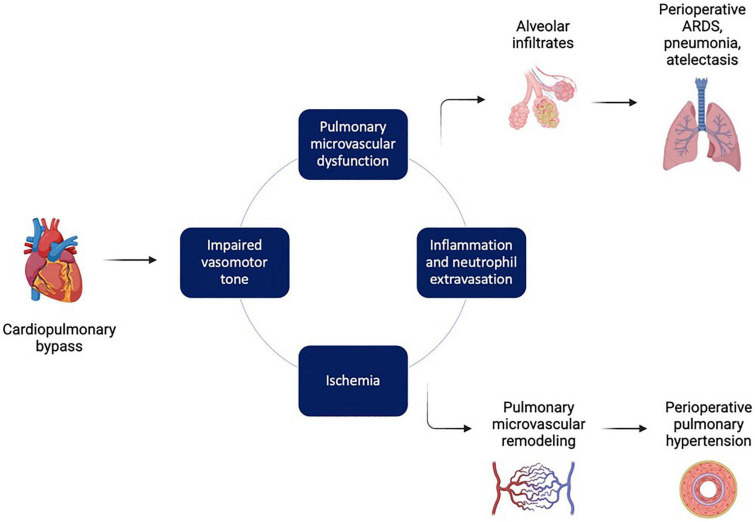
Factors leading to pulmonary injury during and after cardiopulmonary bypass. Cardiopulmonary bypass (CPB) triggers a pathological series of impaired vasomotor tone, ischemia, and inflammation that drives pulmonary microvascular dysfunction. This in turn can lead to disruption of the alveolar-capillary membranes, pulmonary edema, acute respiratory distress syndrome (ARDS), and atelectasis. In addition, pathologic pulmonary microvascular remodeling can lead to postoperative pulmonary hypertension. Figure created using BioRender.com.

A variety of pulmonary complications may occur after cardiac surgery (Figure 5). One observational analysis of 517 patients undergoing cardiac surgery involving CPB reports a postoperative pulmonary complication incidence of 6.2%, including atelectasis respiratory failure, pneumonia, and acute respiratory distress syndrome (ARDS) (189). Retrospective analysis found that major risk factors for postoperative pulmonary complications included advanced age (> 60 years), prolonged bypass time, and preoperative pulmonary hypertension (189). Similarly, a prospective study examining 179 cardiac surgery patients found a cumulative pulmonary complication incidence of 15.08%; more specifically, in 7.82% of CABG patients, 2.23% of valve replacement patients, and in 5.05% of patients with correction of congenital heart disease (190).

#### 3.3.2. Causes of pulmonary microvascular dysfunction following CPB

Pulmonary vascular dysfunction may contribute to lung injury following cardiac surgery. Microvascular inflammation and ischemia-reperfusion are major contributors to postoperative ARDS, a highly fatal postoperative pulmonary complication. A retrospective study of 457 patients undergoing valvular surgery showed that 8.1% of patients developed postoperative ARDS, with a mortality rate of 29.7% (191). Furthermore, more severe stages of ARDS were associated with reduced postoperative survival (191).

Pro-inflammatory cytokines, chemokines, and complement activated by the systemic inflammatory response to cardiac surgery activate endothelial cells in the pulmonary vasculature (192). As discussed earlier, activated endothelial cells upregulate adhesion molecules on their luminal surface, facilitating leukocyte rolling, adhesion, and transmigration across the pulmonary endothelium (192). Increased endothelial barrier permeability after cardiac surgery, in part due to inflammation-induced endothelial injury, further potentiates extravasation of vascular luminal contents (193, 194). This process results in activated neutrophils and macrophages entering the lung parenchyma and secreting ROS and matrix metalloproteinases that cause alveolar damage and further promote influx of proteinaceous fluid into damaged alveoli (195).

As with the renal and cerebral microcirculations, ischemia-reperfusion also contributes to microvascular injury in the pulmonary microcirculation. During CPB, the lungs are solely supplied by the bronchial circulation, with paradoxical pulmonary vasoconstriction leading to decreased bronchial arterial flow and relative pulmonary ischemia (196, 197). A porcine model of CPB showed that bronchial artery ligation prior to initiation of CPB resulted in markedly increased signs of lung injury compared to no bronchial artery ligation (198). Decreased bronchial artery blood flow during and after CPB resulted in increased pulmonary edema and significant intrapulmonary shunting that in turn led to severe arterial hypoxemia (198). Finally, it is also plausible that increased inflammatory activity in pulmonary capillaries restricts area available for adequate gas exchange and further disrupts integrity of the alveolar-capillary barrier, contributing to postoperative pulmonary edema, atelectasis, and ARDS.

Cessation of pulmonary arterial clamping in CPB allows for reperfusion, which then triggers reperfusion injury driven by increased oxidative stress in response to sudden oxygen overload after a period of prolonged ischemia (196). Furthermore, the particularly small diameter of pulmonary capillaries may lead to activated neutrophils and platelets impeding red blood cell transit, obstructing the microcirculation, and resulting in persistent vascular insufficiency even after reperfusion (196). Inflammation and ischemia-reperfusion work in combination to cause microvascular dysfunction, disrupt endothelial and alveolar epithelial barriers, and facilitate postoperative ARDS, along with other pulmonary complications such as atelectasis and pleural effusions.

#### 3.3.3. Pulmonary microvascular dysfunction and postoperative pulmonary hypertension

Microvascular dysfunction in the pulmonary system may also contribute to postoperative pulmonary hypertension. The Sixth World Symposium in Pulmonary Hypertension defined pulmonary hypertension as a mean pulmonary arterial pressure (mPAP) greater than 20 mmHg (199). Currently, there are five defined categories of pulmonary hypertension: pulmonary arterial hypertension (an idiopathic hypertensive vasculopathy of the pulmonary arteries), pulmonary hypertension due to left-sided heart disease, pulmonary hypertension due to lung disease or hypoxia, pulmonary hypertension due to pulmonary artery obstruction, and pulmonary hypertension of unknown etiology (199). Over time, prolonged pulmonary hypertension promotes pulmonary vascular remodeling that leads to further elevations in pulmonary vascular pressures, ultimately resulting in right heart failure and significant morbidity and mortality (200).

Pulmonary hypertension after cardiac surgery may result from several complex mechanisms acting in concert, including preoperative structural cardiac dysfunction, altered vasomotor tone, inflammation, and ischemia-reperfusion injury (201). First, preoperative left ventricular systolic or diastolic dysfunction, along with valvular disease, may predispose patients to increased risk of pulmonary hypertension after cardiac surgery involving cardiopulmonary bypass (201). Next, altered vasomotor tone in the pulmonary microcirculation following CPB in animal models has been well-characterized in several studies. Impaired cholinergic and beta-adrenergic responses, impaired endothelium-dependent vasodilation, increased endothelin-1 responsiveness, and impaired nitric-oxide-induced vasodilation together result in increased pulmonary vasoconstriction, decreased myogenic tone, and increased pulmonary vascular resistance (3, 56, 202–208). Of note, administration of protamine during cardiac surgery can also cause significant pulmonary vasoconstriction through a mechanism involving reduced nitric oxide release in the pulmonary vasculature (209, 210).

Dora et al. studied isolated human pulmonary intralobular arteries through lung biopsies from patients undergoing CABG surgery and found significantly altered pulmonary arterial contractile tone and endothelium-dependent vasodilation postoperatively (211). Furthermore, live cell viability analysis found that while 90% of harvested vascular endothelial cells had intact cell membranes preoperatively, only 58% had intact cell membranes postoperatively (211). Inflammation and ischemia during cardiac surgery may also contribute to pulmonary hypertension through pulmonary vascular remodeling driven by ROS products, such as isoprostanes, and dysregulated growth factor activity (e.g., PDGF-induced pulmonary arterial smooth muscle proliferation) (212–214).

Little to no literature exists specifically investigating the incidence of pulmonary hypertension after cardiac surgery in humans. Results of a swine model showed that CPB resulted in pulmonary hypertension and increased pulmonary vascular resistance (215). Similar results were found in other studies using porcine models (204, 208). Moreover, hypothermic CPB significantly augmented pulmonary vascular resistance and vasoconstriction responses to endothelial agonists in a lamb model (216). Further research examining postoperative pulmonary hypertension in humans are required to better characterize this complication.

Several studies have investigated potential therapeutic targets for postoperative pulmonary hypertension. One study demonstrated that inhaled nitric oxide administered to patients during CPB resulted in lower postoperative mPAPs (217). Administration of inhaled nitric oxide postoperatively after weaning from bypass has also been shown to reduce mPAPs and pulmonary vascular resistance (218, 219). Iloprost leads to decreased mPAPs and pulmonary vascular resistance after weaning from CPB in adults and children (220, 221). Other vasodilatory drugs that have shown promise include inhaled prostacyclin (219, 222), and the phosphodiesterase inhibitors, sildenafil (223–225) and milrinone (226, 227), particularly after mitral valve operations.

Of note, longer duration of CPB has been linked with increased incidence of postoperative lung injury; however, the mechanisms are unclear. One possibility may be extended restriction of bronchial artery blood circulation, which is substantially reduced during CPB and has been associated with pathologic metabolic and ultrastructural changes in lung tissue (228). Alternatively prolonged CPB time may promote a prolonged intraoperative inflammatory response in the pulmonary system and more extensive coagulation cascade activation in the pulmonary microcirculation, restricting nutrient and gas exchange and disrupting the integrity of the alveolar-capillary interface (228, 229).

#### 3.3.4. Impact of pre-existing pulmonary microvascular dysfunction

As in the renal and brain systems, pre-existing comorbidities may influence extent of postoperative microvascular dysfunction following CPB in the pulmonary system. For example, recent studies suggest a link between both type 1 and type 2 DM and pulmonary arterial disease (230). Indeed, patients with type 2 DM appear to have a higher prevalence of pulmonary hypertension, which is independent of hypertension, coronary artery disease, or heart failure (230). Furthermore, streptozotocin-induced type 1 DM rats exhibited extensive pulmonary endothelial dysfunction associated with increased superoxide production and enhanced pulmonary vascular responsiveness to serotonin (230, 231).

Moreover, an analysis of over 200 patients with pulmonary hypertension showed that patients with diabetes trended toward reduced responses to inducible nitric oxide, a finding consistent with animal models of DM that showed reduced pulmonary vascular tone and responsiveness to vasodilators (232). Unfortunately, few studies have investigated the impact of pre-existing DM on postoperative lung injury following cardiac surgery. One study by Lauruschkat et al. found that patients with undiagnosed and insulin-treated DM have higher risk of postoperative pulmonary complications than non-diabetic patients; however, the specific mechanisms behind this finding are unclear (233).

#### 3.3.5. Techniques of perfusion and pulmonary microvascular injury

Finally, several studies have examined the impact of pulsatile vs. non-pulsatile CPB on postoperative lung injury following CPB. A study of patients with chronic obstructive pulmonary disease (COPD) undergoing cardiac surgery showed that pulmonary vascular resistance at the time of aortic cross clamping was significantly lower in the pulsatile vs. non-pulsatile group, along with duration of postoperative mechanical ventilation (234). Next, in a porcine model of CPB, pulsatile perfusion was associated with a reduced pulmonary inflammatory response, evidence by lower levels of NF-kappa-beta, IL-1, IL-6, and TNF-alpha (235). Moreover, a meta-analysis of 8 studies involving 474 patients receiving pulsatile perfusion vs. 496 patients receiving non-pulsatile perfusion during CPB found that patients receiving pulsatile perfusion had significantly lower incidences of postoperative ARDS and intubation time (236).

Santini et al. report that pulsatile pulmonary perfusion during CPB in CABG patients resulted in better preserved alveolar-arterial oxygen gradients, lung compliances, and overall pulmonary hemodynamics (237). The authors also found that postoperative bronchoalveolar lavage specimens from patients undergoing pulsatile perfusion demonstrated lower absolute white counts and lower percentage of neutrophils (237). Donodov et al. found a similar benefit of pulsatile perfusion with respect to pulmonary protection during cardiac surgery; however, Engels et al. found no significant differences in postoperative ventilation time between pulsatile vs. non-pulsatile groups (238, 239).

Altogether, pulmonary microvascular injury during cardiac surgery due to inflammation, altered vasomotor tone, and ischemia-reperfusion can lead to several adverse effects in the lungs including postoperative ARDS, atelectasis, and (if extensive pulmonary microvascular remodeling occurs), postoperative pulmonary hypertension.

### 3.4. Vascular dysfunction: Impact on peripheral circulations

While most research has focused on examining vascular damage following cardiac surgery in major organ systems such as lungs, brain, and kidneys, a smaller body of studies has considered the impact of cardiac surgery on certain peripheral microcirculations, such as skin and mucous membranes. It is important to consider the peripheral circulation due to its critical role in transporting blood throughout the body, storing blood, promoting exchange between blood and tissue, and regulating cardiac output (240). One such study assessed endothelium-dependent microvascular reactivity in the skin of the foreheads of children and infants undergoing correction for cyanotic and acyanotic congenital heart disease, using single point laser Doppler perfusion monitoring and local thermal hyperemia to examine blood flow (241). Vasodilation induced by local thermal hyperemia was significantly attenuated during CPB relative to preoperative responses, and these responses largely normalized after cessation of bypass (241).

An earlier study using laser Doppler monitoring of cutaneous vessels before, during, and after CABG in adults had shown similar findings of reduced postoperative cutaneous blood flow, which improved by postoperative day 6 (242). Finally, several studies have demonstrated reactive hyperemia in cutaneous microvessels following CPB, such as a study by Gruber et al. examining forearm blood flow, and a study by Pezawas et al. that used skin surface temperature gradients as a proxy for microvascular tone (243, 244).

Hence cardiac surgery can have a significant impact on peripheral microvascular reactivity in the postoperative period.

## 4. Conclusion

A variety of mechanisms contribute to microvascular dysfunction following cardiac surgery. Cardiac surgery significantly disrupts the balance between vasodilators and vasoconstrictors in the microcirculation through altered responsiveness to endothelium-derived and neuromodulatory vasoactive mediators. This leads to altered vasomotor and myogenic tone, and increased propensity for vasospasm that disrupts normal tissue perfusion. Additionally, CPB induces a systemic pro-inflammatory state characterized by endothelial activation, activation of coagulation and complement pathways, arachidonic acid metabolism, and generalized endothelial injury throughout the microvasculature. Microvascular dysfunction in turn leads to organ damage in several key systems. Hypoperfusion, ischemia-reperfusion, and microvascular inflammation are common themes underpinning postoperative renal, brain, and pulmonary injury. Altogether, understanding the effects of cardiac surgery on microvascular function and organ damage will prove crucial for future innovators working to improve surgical technique and develop new methods of organ protection to reduce postoperative morbidity and mortality.

## Author contributions

JF contributed to conceptualization, design, and revision of the manuscript. SK and DB contributed to drafting, design, and revision of the manuscript. SS contributed to design and revision of the manuscript. FS contributed to revision of the manuscript. All authors contributed to the article and approved the submitted version.
